# Factors associated with low birthweight in North Shewa zone, Central Ethiopia: case-control study

**DOI:** 10.1186/s13052-018-0516-7

**Published:** 2018-07-04

**Authors:** Berhanu Gizaw, Samson Gebremedhin

**Affiliations:** 1 Department of Public Health, Selale University, Salale, Ethiopia; 2 School of Public Health, Hawassa University, Hawassa, Ethiopia

**Keywords:** Low birthweight, Risk factors, Case-control study, North Shewa zone, Ethiopia

## Abstract

**Background:**

Low birthweight (LBW) is an important predictor of neonatal and post neonatal child morality. Though its risk factors have been extensively studied in the developed world; limited epidemiological evidence is available in developing countries including Ethiopia. The purpose of the study is to determine the risk factors of LBW in North Shewa zone, Central Ethiopia.

**Methods:**

Unmatched case-control study involving 94 cases and 376 controls was conducted from Jan to Mar 2017 in three public hospitals in the zone. A case was defined as a singleton live birth with birthweight less than 2.5 kg; whereas, a control was a newborn that weighs 2.5–4.0 kg. Cases and controls were recruited on an ongoing basis until the required sample sizes were fulfilled. Data were collected by interviewing mothers, reviewing medical records and measuring the anthropometry of the mothers and the newborns. Bivariable and multivariable logistic regression analyses were used to identify risk factors of LBW. The outputs of the analyses are presented using adjusted odds ratio (AOR) with the respective 95% confidence interval (CI).

**Results:**

Mothers with no formal education had two times increased odds of delivering LBW babies than women with formal education [AOR = 2.20 (95% CI: 1.11, 4.38)]. Mothers with no history of nutrition counseling during pregnancy had three times increased odds of giving LBW babies than those who were counseled [AOR = 3.35 (95% CI: 1.19, 9.43)]. Non-married women had higher odds of giving LBW newborns as compared to married ones [AOR = 3.54 (95% CI: 1.83, 6.83)]. Mothers from food insecure households had about four times higher odds of LBW as compared to food secure mothers [AOR = 4.42 (95% CI: 1.02,22.25)]. In contrast to mothers who had the recommended four or more antenatal care (ANC) visits, those who were not booked had three times increased odds of giving to LBW baby [AOR = 3.03 (95% CI: 1.19,7.69)].

**Conclusion:**

Improving the socio-economic status of mothers, enhancing the utilization of ANC and strengthening the integration of nutrition counseling into ANC help to reduce LBW.

## Background

According to the definition of the World Health Organization (WHO), low birthweight (LBW) is weight of an infant at birth of less than 2500 g irrespective of the gestational age [1]. LBW can result from premature birth (before 37 weeks of gestation) or intrauterine-growth restriction (IUGR), or a combination of the two [1, 2]. In the developing world, the main cause of LBW is assumed to be IUGR; whereas in developed countries prematurity has higher significance [1, 2].

Birthweight is the single most important predictor neonatal and post-neonatal survival. It has been estimated that LBW babies are approximately 20 times more likely to die than normal babies in the first year of life [3]. LBW babies are also at increased risk of developing long-term sequels including cognitive developmental delays and decrease of intelligence quotient (IQ) scores [4–7]. The fetal origins hypothesis also suggests that LBW raises the risk of developing some non-communicable diseases later in adulthood life [8–10].

It is estimated that globally 15to 20% of all births are LBW, representing more than 20 million births a year [1]. There is considerable variation in the magnitude of LBW across regions and within countries [1]. More than 95% of LBW babies – 72% in Asia and in 22% in Africa – are born in developing countries [1]. The regional estimates of LBW showed that Sub-Saharan Africa is the second, with the regional average of 13% next to South Asia (28%) [1, 11]. Hospital-based studies cross-sectional studies conducted in Ethiopia in the last 10 years reported prevalence of LBW ranging from 6 to 23% [12–17]. On the other hand, community-based prospective studies came-up with 9–28% prevalence [18–22]. In 2012 the United Nations Children’s Fund (UNICEF) estimated the national prevalence of LBW was 20% [23].

LBW is caused by multifold and possibly overlapping factors that affect either the duration of pregnancy or fetal growth, or both [2, 3]. According to a systematic review [3], the risk factors of LBW can be broadly classified as direct and indirect. Indirect factors include socio-demographic and economic features and maternal age; whereas direct factors comprise: race, maternal height and pre-pregnancy weight, gestational weight gain and calorie intake during pregnancy, perinatal morbidity, paternal anthropometry, parity, infant’s sex, alcohol and cigarette exposure during pregnancy and prior history of prematurity or IUGR [3].

Though the risk factors of LBW have been extensively studied in the developed world; there is limited epidemiological evidence in developing countries including Ethiopia. The relative significance of the risk factors is also likely to vary across settings secondary to underlying contextual factors. Accordingly this case-control study was designed to identify factors associated with LBW in North Shewa zone hospitals, central Ethiopia.

## Methods

### Study setting

The study was carried out in three secondary-care public hospitals – Fiche, Kuyu and Dera –found in North Shewa zone, Oromia region, central Ethiopia. As of 2016, North Shewa zone had an estimated population size of 1.5 million. The zone is administratively divided in to 13 districts and has the aforementioned 3 functional hospitals, 62 health centers, 268 health posts. In 2016, the total health facility deliveries in the zone were 38,131.

### Study design and period

Unmatched case-control study with controls-to-case ratio of 4:1 was conducted from January 01 to March 30, 2017.

### Study participants and eligibility criteria

Singleton live births in the three hospitals during the study period, irrespective of the duration of pregnancy and mode of delivery, were considered eligible for the study. Birthweight of every child was measured and newborns who weigh less than 2.5 kg were taken as cases; whereas, a similar group of children with a birthweight of 2.5 to 4.0 Kg were categorized as a controls. Multiple births, macrosomic babies (birthweight greater than 4.0 kg), mothers or newborns in critical medical conditions and babies weighed more than an hour after birth were excluded.

### Sample size determination and sampling approach

Optimal sample size was determined via the online OpenEpi statistical program [24]. The computation was made using double population proportion formula assuming 95% confidence level, 80% power, control-to-case quotient of 4, and odds ratio (OR) of 2 to be detected as significant. The calculation was separately made for four potential predictors (maternal age (>/< 18 years), place of residence, birth interval (>/< 2 years) and maternal MUAC (>/< 22 cm)) of LBW and the maximum was taken as the ultimate sample size of the study. The expected proportions of controls exposed for the aformentioned factors were extracted from a study conducted in Southeastern Ethiopia [25]. Ultimately the sample size 94 cases and 376 controls was determined. Between Jan to Mar 2017, cases and controls were recruited on an ongoing basis until the required sample size was fulfilled for both groups.

### Data collection tools and procedures

The data were collected by interviewing the mothers, reviewing medical records and measuring the anthropometry of the mothers and the newborns.

Six trained midwives working in the delivery wards of the three hospitals collect the data using structured and pretested questionnaire prepared in Afan Oromo language. Eligible mothers were interviewed face to face within 24 h after delivery. Socio-demographic and economic information was assessed using standard questions extracted from the DHS questionnaire [26].

The medical records of the mothers were reviewed and relevant information including last-normal menstrual period and ultrasound dating of pregnancy were extracted to the questionnaire.

The frequency of consumption of eleven major food groups during the pregnancy was measured using a Food Frequency Questionnaire (FFQ) based on the mothers’ recall. On the other hand, the level of household food insecurity was assessed using the Household Food Insecurity Access Scale (HFIAS) of the Food and Nutrition Technical Assistance (FANTA) project. The scale categorized the subjects into four ordinal groups – secure; mild, moderate and severe insecurity [27].

The weight of the newborns was measured within the first hour of birth using a calibrated Seca scale and rounded to the nearest 100 g. MUAC of mother was measured to the nearest 0.1 cm using MUAC tape. Anthropometric measurements were taken in duplicates by an observer and ultimately the average of the duplicates was registered.

### Variables of the study

The independent variables of the study include socio-demographic factors (maternal age, education, occupation, wealth index, residence, marital status, religion, ethnicity), reproductive factors (gestational age, prior history of LBW, parity, birth-to-birth interval, utilization of antenatal care (ANC), reported illness during pregnancy), nutritional factors (maternal MUAC, household food security status, exposure to nutrition counseling during the pregnancy, frequency of consumption of major food groups, restriction of diet during pregnancy due to food taboo, history prenatal iron supplementation), work load during pregnancy and infant’s sex. The dependent variable was birthweight status dichotomized into LBW or normal birthwieght.

### Data management and analysis

The collected data were checked for completeness, coded and entered into Epi info version-3.5, and then exported to the Statistical Package for Social Sciences (SPSS), version 20 for analysis.

Wealth index was computed as a composite indicator of living standard based on ownership of selected household assets, size of agricultural land, number of livestock owned, materials used for housing construction, and ownership of improved water and sanitation facilities. The analysis was made using the Principal Component Analysis (PCA). The generated principal component was divided into three wealth classes.

The socio-demographic and other background profiles of the cases and controls were compared using chi-square test. Prior to analysis the assumptions of chi-square test were checked. When smaller expected frequencies were encountered, re-categorization of variables or merger of the levels was made.

Factors associated with LBW were identified using bivariable and multivariable logistic regression models. Independent variables that demonstrated near to statistically significant association (*p*-value less than 0.25) with the outcome variable in the bivariable models, were considered as candidate variables for the multivariable logistic regression models. In order to reduce over adjustment bias, direct and indirect predictors of LBW were fitted separately into two multivariable models [3]. In the ultimate multivariable models the level of multicolinearity was evaluated using variance inflation factor and found within a tolerable range. The goodness-of-fit assessed using Hosmer–Lemeshow test.

### Ethical considerations

The study was approved by the Institutional Review Board (IRB) of College of Medicine and Health Sciences, Hawassa University. Data were collected after taking informed consent from the mothers.

## Results

### Socio-demographic characteristics of the study participants

A total of 470 mother-newborn dyads comprising 94 cases and 376 controls were included in the study. The mean (±SD) age of the mothers of the cases was 27.4 (±6.6) years and that of mothers of the controls was 29.0(±6.4) years. About two-fifth (41.5%) of the mothers of the cases and about a quarter (27.7%) of that of the controls were in the age group 15–24 years and the difference was significant (*p* = 0.027). Higher proportion of the mothers of the cases (53.2%) had no formal education than that of the controls (42.0%) (*p* = 0.002). The two groups were significantly different in terms of ethnicity, religious affiliation and marital status (*p* < 0.05). However, there were no significant variations based on  household wealth index, place of residence and maternal employment status (*p* > 0.05) (Table 1).

**Table 1:** Socio-demographic characteristics of the mothers who gave birth in North Shewa zone hospitals, Central Ethiopia, 2017

Variables	Cases (*n* = 94)		Controls (*n* = 376)		*p*-value
	Number	Percent	Number	Percent	
Maternal age (years)					
15–24	39	41.5	104	27.7	0.027^*^
25–34	38	40.4	200	53.2	
35–49	17	18.1	72	19.1	
Place of residence					
Rural	59	62.8	230	61.2	0.776
Urban	35	37.2	146	38.8	
Maternal educational status					
No formal education	50	53.2	163	42	0.002^*^
Primary school	29	30.9	83	22.1	
Secondary or tertiary education	15	16	130	34.6	
Religion					
Orthodox Christian	62	66	306	81.4	<0.001^*^
Protestant	22	23.4	33	8.8	
Muslim	10	9.6	33	8.8	
Ethnicity					
Oromo	79	84	346	92.0	0.044^*^
Amhara	10	10.6	23	6.1	
Others	5	5.3	7	1.9	
Occupation					
Employed/Merchant	27	28.7	126	33.5	0.376
Housewife	66	70.2	244	64.9	
Others	1	1.1	6	1.6	
Wealth index					
Poorest or poorer	38	40.4	150	39.9	0.911
Middle	20	21.3	74	19.7	
Richer or richest	36	38.3	152	40.4	
Marital status					
Married	73	77.7	346	92.0	<0.001^*^
Others	21	22.3	30	8.0	

*Statistically significant difference at *p*-value of 0.05

### Reproductive characteristics

Regarding the reproductive profile, more cases (16.0%) were preterm births than the controls (5.9%) (*p* = 0.001). Among mothers who had at least two births, short birth-to-birth interval (less than 2 years) was observed in 52.8% of the cases and 21.2% of the controls (*p* < 0.001). Among mothers of the cases, 25.5% had no ANC visits as compared to 13.8% in that of the controls (*p* < 0.001). Significant differences were also observed between the groups in the number of ANC consultations (*p* < 0.001). However, the mothers of cases and controls were not significantly different in terms of parity, presence of pregnancy related complications (*p* = 0.403) and infant’s sex (*p* = 0.781) (Table 2).

**Table 2:** Reproductive characteristics of the mothers who gave birth in North Shewa zone hospitals, Central Ethiopia, December 25, 2016-March 29, 2017

Variables	Cases(n = 94)		Controls(n = 376)		*p*-value
	Number	Percent	Number	Percent	
Gestational age (in weeks)					
< 37	15	16.0	22	5.9	0.001^*^
≥ 37	79	84	354	94.1	
Parity					
Primipara	41	43.6	135	35.9	0.167
Parous	53	56.4	241	64.1	
Birth-to-birth interval					
Less than two years	28	52.8	51	21.1	<0.001^*^
Two or more years	25	47.2	190	78.8	
ANC during the current pregnancy					
No ANC	24	25.5	52	13.8	<0.001^*^
1–3 visits	63	67.1	237	63.1	
4 or more visits	7	7.4	87	23.1	
Trimester at first ANC					
First	8	11.4	26	8.0	<0.001^*^
Second	38	54.3	273	84.3	
Third	24	34.3	25	7.7	
Pregnancy complications					
No	86	91.5	353	93.9	0.403
Yes	8	8.5	23	6.1	
Infant’s sex					
Female	45	47.9	174	46.3	0.781
Male	49	52.1	202	53.7	

*Statistically significant difference at *p*-value of 0.05

### Nutrition related characteristics of the study participants

Nearly half (44.9%) of mothers of the cases and 58.5% of that of the controls received no nutrition counseling during the pregnancy (*p* = 0.019). In contrast to 42.3% of the mothers of the control babies, 28.0% of mothers of the cases had taken prenatal iron supplements (*p* = 0.009). About half (52.1%) of the households of the controls were food secure but the corresponding figure was significantly lower (26.6%) in the cases group (*p* < 0.001). The two groups were not significantly different based on reported practice of food taboo during pregnancy and prevalence of thinness (MUAC less than 210 mm) (Table 3).

**Table 3:** Nutritional and household food security profile of the mothers of the cases and controls, North Shewa zone hospitals, Central Ethiopia, 2017

Variables	Cases (n = 94)		Controls (n = 376)		*p*-value
	Number	Percent	Number	Percent	
Restriction of diet due to food taboos					
No	89	94.7	368	97.9	0.091
Yes	5	5.3	8	2.1	
Nutrition counseling during pregnancy					
Yes	39	41.5	207	55.1	0.019^*^
No	55	58.5	169	44.9	
Use of iron tablets in recent pregnancy					
No	68	72.3	217	57.7	0.009^*^
Yes	26	27.7	159	42.3	
MUAC of mother (mm)					
< 210	36	38.3	122	38.4	0.283
≥ 210	58	61.7	254	67.6	
Household food security status					
Secure	25	26.6	196	52.1	<0.001^*^
Insecurity	69	73.7	180	47.9	

*Statistically significant difference at *p*-value of 0.05

The overall dietary intake of the mothers was assessed using a FFQ. However, the cases and controls were not significantly different in the frequency of consumption of 11 food groups. The food groups which considered in the study were: cereals, roots and tubers, legumes, milk and milk products, flesh foods, eggs, vitamin A rich fruits and vegetables, other fruits, other vegetables, sweets and condiments.

### Risk factors of low birthweight

As it was stated earlier, risk factors of LBW were identified by fitting two different multivariable regression models for the direct and indirect predictors of birthweight. The variables were classified into the two blocks according to the Kramer’s framework [3].

For the indirect model, six socio-demographic variables (maternal age, place of residence, maternal education, occupation of the mother, household wealth index, religion, ethnicity and marital status) were considered. Among these, based on bivariable logistic regression analyses, six variables presented in Table 5 had *p*-value less than 0.25 hence fitted into the multivarible model.

The ultimate analysis showed the odds of LBW were increased by two fold in the mothers with no formal education as compared to their counterparts. Those who were not married had three times elevated odds of LBW as compared to married mothers. Protestant Christians had three times increased odds of LBW than Orthodox Christians (Table 4).

**Table 4:** Socio-demographic factors associated with low birthweight among deliveries in North Shewa zone hospitals, Central Ethiopia, 2017

Variables	Cases	Controls	COR (95% CI)	AOR (95% CI)
	(n = 94)	(n = 376)		
Age in years				
15–24	39	104	1.59 (0.83,3.02)	0.54 (0.27–1.06)
25–34	38	200	0.81 (0.43,1.51)	1.21 (0.60–2.46)
35–49	17	72	1^r^	1^r^
Residence				
Rural	59	230	1^r^	1^r^
Urban	35	146	0.94 (0.59,1.49)	0.66 (0.16–2.70)
Educational status				
No formal education	50	163	0.88 (0.52,1.49)	2.20 (1.11–4.38)*
Primary school or above	29	83	1^r^	1^r^
Occupation				
Employed /merchant	27	126	1^r^	–
Housewife	66	244	1.26 (0.77,2.07)	–
Wealth index				
Poorest or poorer	38	150	1.07 (0.64,1.78)	–
Middle	20	74	1.14 (0.62,2.11)	–
Richer or richest	36	152	1^r^	–
Marital status				
Married	73	346	1^r^	1^r^
Others	21	30	3.32 (1.80,6.12)*	3.54 (1.83–6.83)*
Religion				
Orthodox Christian	62	306	1^r^	1^r^
Muslim	10	33	1.50 (0.70–3.19)	1.23 (0.54–2.81)
Protestant	22	33	3.29 (1.80–6.02)*	3.43 (1.73–6.80)*
Ethnicity				
Oromo	79	346	0.52 (0.24–1.15)	0.53 (0.24,1.15)
Others	5	7	1.64 (0.42–6.44)	1.64 (0.42,6.45)
Amhara	10	23	1^r^	1^r^

1^r^ Reference group; * Significant association at *p*-value of 0.05; -The variable was not included in the multivariable model

For the direct model, ten variables (namely gestational age, parity, number of ANC visits, presence of pregnancy related complications, infant’s sex, restriction of diet due to food taboos, dietary counseling in the index pregnancy, use of iron supplements in the pregnancy, MUAC of the mother, and household food security status) were considered. Among them, based on bivariable analysis, five variables presented in Table 5 had *p*-value less than 0.25 and hence subjected to the multivariable analysis.

**Table 5:** Reproductive and nutrition related factors associated with low birthweight, North Shewa zone hospitals, Central Ethiopia, 2017

Variables	Cases	Controls	COR (95% CI)	AOR (95% CI)
	(n = 94)	(n = 376)		
Gestational age				
< 37 weeks	15	22	3.06 (1.52,6.15)*	3.70 (0.42,33.33)
≥ 37 weeks	79	354	1^r^	1^r^
Parity				
Primiparous	41	135	1.38 (0.87,2.19)	–
Multiparous	53	241	1^r^	–
ANC during the current pregnancy				
No ANC	24	52	5.73 (2.31,14.24)*	3.03 (1.19,7.69)*
1–3 visits	63	237	3.30 (1.46,7.49)*	3.13 (0.99,9.10)
4 or more visits	7	87	1^r^	1^r^
Presence of pregnancy complications				
No	86	353	0.72 (0.31,1.66)	–
Yes	8	23	1^r^	–
Infant’s sex				
Female	45	174	1.07 (0.68,1.68)	–
Male	49	202	1^r^	–
Avoidance of food due to food taboos				
No	89	368	0.39 (0.12,1.21)	0.31 (0.06,1.58)
Yes	5	8	1^r^	1^r^
Dietary counseling during pregnancy				
Yes	39	207	1^r^	1^r^
No	55	169	1.73(1.09,2.73)*	3.35(1.19,9.43)*
Use of iron tablets				
No	68	217	1^r^	1^r^
Yes	26	159	0.52 (0.32,0.86)	0.58 (0.19,1.78)
MUAC of mother (mm)				
< 210	36	122	1.29 (0.81,2.06)	–
≥ 210	58	254	1^r^	–
Food security status				
Secure	25	196	1^r^	1^r^
Insecure	69	180	3.01 (1.82,4.96)*	4.42 (1.02,22.25)*

1^r^ Reference group; * Significant association at *p*-value of 0.05; -The variable was not included in the multivariable model

The ultimate model showed that mothers who have no history of nutrition counseling during the pregnancy had more than three folds increased odds of delivering LBW babies than those who got counseling. Mothers from food insecure households had about four times higher odds of LBW as compared to food secure mothers. Taking mothers who had the recommended four or more ANC visits as reference, those who were not booked had three times increased odds of giving to LBW baby (Table 5).

## Discussion

This study identified socio-demographic, reproductive and nutrition related risk factors of LBW. From socio-demographic factors, absence of formal maternal education and unmarried marital status were significant predictors of LBW. Further, mothers who did not receive nutrition counseling during pregnancy and those who were from food insecure households were at increased odds of LBW.

We found that mothers with no formal education had increased odds of giving to LBW newborns. This is parallel to the findings of studies conducted South-East Ethiopia [25], rural Sidama, Southern Ethiopia [18], India [28, 29] and Tanzania [30]. This can be explained by the fact that formal education enables women to improve their capacity to generate income and to promote optimal dietary practices during pregnancy. Further it may also enhance their awareness about other risk factors of LBW. Education enables women to make independent decisions and to have better access to household resources that are important for better nutrition [31].

Being married was identified as a protective factor from LBW. Reasonable number of studies witnessed that the general health of married women is better than that of unmarried ones [32, 33]. Unmarried women may experience higher stress than married mothers because of less stable relationships. Further married mothers may get socio-economic supports from their husbands so that they will not be under such pressure. This finding is in agreement with a study conducted in Tanzania which observed that unmarried mothers were almost two times more likely to give birth to LBW neonates [31]. A systematic review concluded unmarried mothers have significantly higher risks of LBW and preterm births [34].

The analysis indicated that Protestant Christians had three times increased odds of LBW than Orthodox Christians. Though religious affiliation can theoretically affect dietary habits and food restrictions during pregnancy, we are not aware of the presence of such variations between the two groups in the area. The observed association possibly might have emanated from residual confounding from socioeconomic differences between the groups. Further, as the number of Protestants in the analysis was relatively small, chance could also explained the association.

In this study, the odds of delivering LBW babies were higher among mothers who did not receive nutrition education during the pregnancy as compared to their counterparts. Previous studies have indicated that nutrition education and counseling during pregnancy benefits in reducing preterm and LBW births [35]. An interventional study in Bangladesh demonstrated that regular prenatal nutrition counseling enhances maternal weight gain during the third trimester and increases birthweight of the newborn by 0.4 kg [36]. This may imply that stronger integration of nutrition education into perinatal care may contribute to reducing the risk of LBW.

We observed that food insecure mothers had four fold increased odds of giving birth to LBW babies than their counterparts. Intuitively, food insufficiency increases risk LBW by compromising maternal nutrient intake, pre-pregnancy weight and gestational weight gain rate. A similar pattern of association has been appreciated by studies conducted in Ethiopia and abroad [37, 38]. For instance, a case-control study in Addis Ababa, Ethiopia concluded that mothers in food insecure household were 3.6 times more likely to have LBW newborns [37].

The results suggest that frequent ANC reduces the risk of LBW. The benefit of ANC could emanate from its various components including prevention and management of anemia and other pregnancy complications. Previous studies came up with conflicting findings. A systematic review concluded that prenantal care prevents neither preterm birth nor IUGR [39]. On the other hand, preterm delivery which is more common in mothers having LBW babies may limit the number of late ANC visits and induce spurious association between ANC and LBW.

The findings of the study should be interpreted in consideration of the following methodological shortcomings. As the study employed a retrospective design, recall errors in the measurement of exposures (e.g. frequency of consumption of different food groups or events related to household food insecurity) are possible. Such errors are likely to produce misclassification of exposures and ultimately may underestimate the strength of associations. Further, as any observational study, residual confounding from unmeasured or misclassified variables cannot be ruled out.

For some of the potential risk factors including gestational age, practice of food taboo and occurrence of pregnancy complications, the observed frequencies were low and this might have reduced the statistical power to detect actual differences. Further, due to the case-control nature of the study, we did not measure some potentially predictors of LBW including prepregnancy weight, gestational weight gain and paternal anthropometry. As study was restricted to hospitals the findings may not be fully generalizable to the entire births in the locality.

## Conclusion

The study demonstrated that maternal educational status, marital status, exposure to nutrition counseling during pregnancy and household food insecurity were significant predictors of LBW. Improving the socio-economic status of mothers, expansion of the utilization of ANC and strengthening the integration of nutrition counseling into ANC help to reduce LBW.

## Abbreviations

ANC: Antenatal care
AOR: Adjusted odds ratio
CI: Confidence interval
COR: Crude odds ratio
DHS: Demographic and health survey
FANTA: Food and nutrition technical assistance
FFQ: Food frequency questionnaire
HFIAS: Household food insecurity access scale
IQ: Intelligence quotient
IRB: Institutional review board
IUGR: Intrauterine growth restriction
LBW: Low birthweight
MUAC: Mid-upper arm circumstance
PCA: Principal component analysis
SPSS: Software package for social sciences
UNICEF: United Nations Children’s Fund
WHO: World Health Organization
